# Upregulation of glycosaminoglycan synthesis by Neurotropin in nucleus pulposus cells via stimulation of chondroitin sulfate N-acetylgalactosaminyltransferase 1: A new approach to attenuation of intervertebral disc degeneration

**DOI:** 10.1371/journal.pone.0202640

**Published:** 2018-08-27

**Authors:** Daisuke Sakai, Tomoko Nakai, Shunsuke Hiraishi, Yoshihiko Nakamura, Kiyoshi Ando, Mitsuru Naiki, Masahiko Watanabe

**Affiliations:** 1 Department of Orthopaedic Surgery, Surgical Science, Tokai University School of Medicine, Isehara, Kanagawa, Japan; 2 Research Center for Regenerative Medicine and Cancer Stem Cell, Tokai University School of Medicine, Isehara, Kanagawa, Japan; 3 Institute of Bio-active Science, Nippon Zoki Pharmaceutical Company, Limited, Kato, Hyogo, Japan; SERGAS and IDIS, SPAIN

## Abstract

It is suggested that most cases of low back pain are related to degeneration of intervertebral discs. Disc degeneration is a chronic and progressive disease and the search for effective medical treatments continues. Neurotropin is widely used in Japan and China to treat low back pain and neck–shoulder–arm syndrome. The present study aimed to investigate the effect of Neurotropin on glycosaminoglycan synthesis in nucleus pulposus cells. Cultured human nucleus pulposus cells were treated with Neurotropin every second day for two weeks. Production of glycosaminoglycan was assessed using a dimethyl-methylene blue assay and PicoGreen was used to measure DNA content. Microarray analysis, real-time PCR, and western blotting were performed to assess the biological processes related to Neurotropin-stimulated glycosaminoglycan synthesis. The results showed that the level of glycosaminoglycan normalized to DNA content was significantly upregulated by the addition of Neurotropin. Gene expression profiling showed over two-fold upregulation of 697 genes in response to Neurotropin treatment. Among these genes, ontological analysis suggested significant implication of phosphatidylinositol 3-kinase signaling, and analysis focused on this pathway demonstrated marked upregulation of angiopoietin 1 and insulin-like growth factor 1. Activation of phosphorylation of the signal transducer protein AKT was detected by western blotting. Of the genes related to sulfated glycosaminoglycan synthesis, the greatest increase in mRNA levels was observed for chondroitin sulfate N-acetylgalactosaminyltransferase 1, an enzyme initiating synthesis of chondroitin sulfate side chains attached to a core protein of aggrecan, which is a predominant disc matrix component. These findings suggest that Neurotropin may activate the phosphatidylinositol 3-kinase–AKT pathway and stimulate glycosaminoglycan synthesis through upregulation of expression of mRNA for chondroitin sulfate N-acetylgalactosaminyltransferase 1. Because there was no cytotoxic cellular growth inhibition, Neurotropin treatment might offer an accessible therapeutic strategy for intervertebral disc degeneration.

## Introduction

A 2017 systematic review assessed the available pharmacologic therapies for low back pain (LBP) and reported that the benefits were short-term (<3 months) and that improvements in pain score [1] were small to moderate [2]. LBP is one of the most common conditions in clinical practice and in chronic cases may cause significant morbidity, disability, and lost productivity [3]. It has been suggested that intervertebral disc (IVD) degeneration is one of the major causes of LBP [4,5]. Disc degeneration is a chronic and progressive disease, for which no ideal medical treatment is currently available;in advanced cases, surgical intervention is required for symptomatic relief [6,7]. The pathophysiology of discogenic LBP is suggested to involve single or multiple factors, including sensitization from ingrowth of sensory nerve fibers into the degenerate IVD, inflammation caused by increased production of pathogenic cytokines, and mechanical hypermobility [8]. Furthermore, the biological alterations underlying disc degeneration and the relatively inhospitable biochemical environment within the IVD make it difficult to design new therapeutic solutions [9].

IVDs have three distinct tissue structures: the central gelatinous nucleus pulposus (NP), rich in proteoglycans and collagen type II, the surrounding fibrocartilaginous annulus fibrosus (AF), rich in collagen type I, and the superior and inferior cartilaginous endplates [4,5]. A study examining 25 whole lumbar spine specimens harvested from fresh cadavers reported that that the water and glycosaminoglycan (GAG) content of the NP decreased significantly with aging and with increasing grade of IVD degeneration [10]. Similarly, we previously reported a marked decrease in the number of NP progenitor cells in herniated NP tissues with aging and increasing grade of degeneration [11]. These changes may lead to a loss of structural integrity in the IVD and result in innervation, inflammation, and hypermobility associated with inability of the affected spinal segments to withstand load or other motions [8]. An understanding of the molecular and cellular events in the IVD tissues suggests that one approach to slow or reverse the degenerative process may be regional stimulation of cell proliferation and matrix production; i.e., stimulation of proteoglycan synthesis in the NP to increase swelling pressure, and collagen synthesis in the AF to repair structural defects [12].

Neurotropin® (NTP), a nonprotein extract from inflamed rabbit skin inoculated with vaccinia virus, has been widely used in Japan and China as an analgesic drug for treatment of chronic pain, such as LBP, postherpetic neuralgia, neck–shoulder–arm syndrome, hyperesthesia of subacute myelo-optic neuropathy, and fibromyalgia [13–15]. Its pharmacological and clinical efficacy has also been reported for various conditions including headache, lower extremity pain after epidural anesthesia, and neurotoxicity during anti-cancer therapy [16–21]. The major analgesic effect of NTP is considered to be mediated by activation of descending pain-inhibitory pathways via the serotonergic and noradrenergic systems projecting from supraspinal sites to spinal dorsal horns [22,23]. In addition, NTP has been demonstrated to have anti-inflammatory effects on human NP cells by reducing interleukin (IL)-1β-induced upregulation of cyclooxygenase-2 (COX-2) and tumor necrosis factor-α (TNF-α) *in vitro* [14]. As a component of the arachidonate cascade, COX-2 has been shown to induce production of prostaglandin-E2, which is capable of causing pain [24]. Therefore, this anti-inflammatory function of NTP might be a factor in its analgesic effect on discogenic LBP, because NP cells, AF cells and recruited macrophages produce pro-inflammatory cytokines such as COX-2, TNF-α and IL-6 within the degenerate IVD [8,14]. Despite the various modulating roles of NTP, its anabolic effects have not been well [25].

Based on the hypothesis that addition of NTP to the culture medium of NP cells may enhance proteoglycan synthesis to halt progressive IVD degeneration, we assessed the effect of NTP on regulation of disc matrix production by human NP cells *in vitro*. The predominant disc matrix components are proteoglycans, particularly chondroitin sulfate glycosaminoglycans (CS-GAG), which give the disc its resilient compressive strength by absorbing water [26,27]. Therefore, we assessed the net production of GAG by cultured NP cells treated with NTP and the change in expression of aggrecan, a molecule that is characterized by the presence of approximately 100 GAG chains attached covalently to a core protein [7]. Interestingly an inhibitory effect on growth of neurites by aggrecan extracted from human IVD tissues has been reported. The extract incorporated into tissue culture substrata inhibited the growth of neurites from a human neuronal cell line, SH-SY5Y in a concentration-dependent manner. However, enzymic pretreatments to reduce the glycosylation of disc aggrecan partially abrogated their inhibitory effects [28]. Thus, GAG chains in aggrecan may play a role as barriers to sensory innervation into the IVD, which associates with pain.

In our previous study we showed immunofluorescence staining of collagen type I and elastin produced by mouse AF cells *in vitro* [29]. We cultured and fixed AF cells on the glass chamber slides, which do not have high background fluorescence. In the current study primary human NP cells were less adhesive onto the glass surfaces, therefore we added L-ascorbic acid phosphate-2-phospate (AsAP) to ensure cellular adhesion. This method enabled us to observe aggrecan on the monolayer cultures. Hence we utilized this monolayer culture condition with addition of AsAP for the other cultures on plastic plates as well to investigate the accumulated GAG throughout this study.

Additionally, we also assessed which biological processes were implicated in NTP-induced GAG synthesis. Recent studies involving cultured articular chondrocytes reported that insulin-like growth factor 1 (IGF1) is the major factor in the serum that stimulates proteoglycan synthesis and that phosphorylation of the cell signaling protein AKT by IGF1 is necessary for this biosynthetic function [30,31]. Thus, to detect any cell signaling evoked in the cultured NP cells by NTP treatment, we performed comprehensive gene expression profiling using microarray and then focused on quantification of key factors by real-time PCR. In addition, we performed western blot analysis of phosphorylation of the key signaling protein. Considering the enormous research cost and effort involved in the discovery, development, and clinical trials of a new drug, it is worth reassessing a currently used drug with an established safety profile as a treatment to attenuate disease progression [32]. Our results demonstrated that treatment with NTP upregulated GAG synthesis in human NP cells isolated from herniated IVD tissue, and that this effect was mediated by the phosphatidylinositol 3-kinase (PI3K)–AKT signaling pathway. Moreover, chondroitin sulfate N-acetylgalactosaminyltransferase 1 (CSGalNAcT-1) was shown to be a major effector of the increase in GAG synthesis induced by NTP treatment of human NP cells.

## Materials and methods

### Chemicals and antibodies

NTP was provided by Nippon Zoki Pharmaceutical Co., Ltd. (Osaka, Japan). The biological activity of NTP was expressed in NTP Units (NU). AsAP was purchased from Wako Pure Chemical (#013–12061, Osaka, Japan). Rabbit polyclonal anti-aggrecan antibody was obtained from Merck (MAB2015, Burlington, MA). Rabbit monoclonal anti-AKT and anti-phospho-AKT (Ser473) antibodies were purchased from Cell Signaling Technology, Inc. (#4691, #4060, respectively, Beverly, MA). Rabbit polyclonal antibodies anti-CSGANACT1 and glyceraldehyde-3-phosphate dehydrogenase (GAPDH) were from abcam (Ab83071, Cambridge UK) and Sigma-Aldrich (G9545, St. Louis, MO). Alexa Fluor 488 goat anti-rabbit IgG was purchased from Thermo Fisher Scientific (A-11070, Waltham, MA). Anti-rabbit antibody conjugated with horseradish peroxidase was purchased from GE Healthcare Life Science (NA9340, Little Chalfont, UK).

### Cell isolation and culture

The study was conducted in accordance with protocols approved by the Experimentation Ethics Committee of Tokai University School of Medicine, including the provision of written informed consent for the use of patient-derived surgical waste material. Human specimens from lumbar IVD herniation were harvested from five patients (three men and two women) aged from 29 to 38 years. The NP and AF were carefully separated, and NP tissues were cut into small pieces and digested with TrypLE Express (#12605–028, Gibco, Carlsbad, CA) for 1 h followed by 0.25 mg/ml Collagenase-P (#11213857001, Roche CustomBiotech, Mannheim, Germany) for 2 h at 37°C. The isolated cells were washed twice with α-minimal essential medium (, α-MEM; #135–15175, Wako Pure Chemical), and seeded at a density of approximately 5 × 10^3^ cells/cm^2^. Cells were cultured in α-MEM supplemented with 10% fetal bovine serum (FBS; #172012, Sigma-Aldrich), 100 U/ml penicillin and 100 mg/ml streptomycin (#15140–122, Gibco) at 37°C, 5% CO_2_ under hypoxic conditions of 2% O_2._ The medium was replaced twice a week and the cells were trypsinized (#25200–072 Gibco) and subcultured before they reached confluency. Cells harvested from third-passage cultures were used for individual experiments.

### Overconfluent cultures stimulated with NTP

NP cells were seeded in 25 cm^2^ flasks at a density of 5× 10^3^ cells/cm^2^ and cultured in 10% FBS, α-MEM overnight prior to NTP addition. The cells were stimulated with NTP dissolved in fresh α-MEM supplemented with 10% FBS and 50 μg/ml AsAP. Since ascorbic acid is instable in solution at 37°C and neutral pH, we used a stable analogue, AsAP which had been developed and has similar activity in tissue culture [33]. The α-MEM itself contains 50 μg/ml sodium ascorbic acid, so the total ascorbic acid concentration after addition of AsAP was 100 μg/ml. The medium was replaced every second day for two weeks. Thereafter, overconfluent monolayer NP cells were evaluated for GAG deposition. Unless stated otherwise, NTP concentrations of 0.1 and 1.0 mNU/ml were used. These setting of the dose is based on the clinical prescriptions as follows; two tablets of NTP (8 NU), twice a day or an intravenous injection of one ampoule (3.6 NU) a day. Therefore, 8 NU is taken by oral administration at a time. In the case of 60 kg of body weight, if sixty percent of the weight is assumed to be of water, concentration of NTP in blood plasma will be 8,000 mNU/36,000 ml, that is, 0.22 mNU/ml. In the case of an injection, it will be 0.12 mNU/ml. The experiments were repeated five times using the cells from five donors.

### Cell proliferation assay

Cells were plated in 96-well plates at a density of 3 × 10^3^ cells/well and allowed to attach. Cells from three donors were used, and five wells were set for each culture condition. NTP concentrations of 0.1 and 1.0 mNU/ml were used. Subsequently, cell numbers were determined on days 3, 5, and 7 using a 3-(4,5-dimethyl-2-thiazolyl)-2,5-diphenyl-2H-tetrazolium bromide (MTT; #5224/500, Wako Pure Chemical) assay [34]. The absorbance at 562 nm was determined using a spectrophotometer (Spectra Max i3, Molecular Devices, Sunnyvale, CA).

### GAG and DNA analysis

Cultured samples were washed with Dulbecco’s phosphate-buffered saline (DPBS; DSBN200, DS-Pharma, Osaka, Japan) and digested at 65°C with 0.5 mg/ml papain (P3125, Sigma-Aldrich) dissolved overnight in a 0.2 M sodium phosphate buffer solution supplemented with 8 mg/ml sodium acetate (#192–01075 Wako Pure Chemical), 4 mg/ml ethylenediaminetetraacetic acid sodium salt dihydrate (E-5134, Sigma-Aldrich) and 0.8 mg/ml L-cysteine (C-7880, Sigma-Aldrich). Sulfated GAG content was measured with a 1,9-dimethyl-methylene blue (DMMB) assay (Blyscan; B1000, Biocolor, Belfast, UK) with chondroitin-6-sulfate (B1000, Biocolor) as the standard, using a spectrophotometer to measure absorbance at 656 nm. DNA quantity was determined using a PicoGreen assay (P11496, Thermo Fisher Scientific) with a spectrophotometer in fluorescence mode with excitation at 480 nm and emission at 520 nm. Each colorimetric measurement was done for three wells in 96 well plates for each group of samples from five donors. NTP concentrations of 0.1 and 1.0 mNU/ml were used.

### Immunofluorescence microscopy and image analysis

NP cells were seeded at a density of 3 × 10^3^ cells/cm^2^ on glass chamber slides (2 cm^2^, #354114, BD Falcon, Corning, NY) and cultured to overconfluence (N = 1). After one and two weeks, samples were fixed in 10% formaldehyde (#11-0735-5, Sigma-Aldrich) and blocked with 3.0% bovine serum albumin (#15260037, Gibco) in PBS followed by staining with the indicated primary antibody (anti-aggrecan, 1:50) at 4°C overnight. Samples were then washed with PBS and stained with Alexa Fluor 488-conjugated secondary antibody. Nuclei were stained with 4′,6-diamidino-2-phenylindole dihydrochloride (DAPI;Vec H-1500, Vector Laboratories, Burlingame, CA). Images were captured using an LSM510 12 META confocal microscope (Carl Zeiss, Oberkochen, Germany). NTP concentrations of 0.1 and 1.0 mNU/ml were used. For AKT phosphorylation image analysis, cells were seeded in 96-well black plates at a density of 2 × 10^3^ cells/well and allowed to attach overnight. Cells were incubated ed in serum free α-MEM for 60 min before being exposed to NTP (0.1,1.0 and 10 mNU/ml) for 15, 30, 45, or 60 min (N = 1). Samples were then fixed with 10% formaldehyde and stained with the indicated primary antibody (1:200). For quantitative image analysis, fluorescence images of 49 sites per well were taken using a 10× objective on an ArrayScan VTI (Thermo Fisher Scientific). The average values for the integrated intensity of each cell in duplicate wells for each condition were used to calculate the mean value per condition.

### Real-time quantitative PCR

After a week in culture with NTP, cells were harvested (N = 5) homogenized in lysis buffer, and total RNA was prepared using an SV Total RNA Isolation System (Z3100, Promega, Madison, WI). For each sample, 2 μg of total RNA was reverse transcribed into cDNA using a High Capacity RNA-to-cDNA kit (#4387406, Applied Biosystems, Foster City, CA). For PCR, 2 μl of cDNA template was used for each reaction, and sequences were amplified using Taq DNA polymerase that had been supplied with a PCR master mix (#4324018, Applied Biosystems). Relative quantification of the target mRNA was performed using the comparative C_T_ method with the endogenous control GAPDH provided as predeveloped TaqMan Assay Reagents by Applied Biosystems. The individual sets of primers and probes (#448892, Applied Biosystems) were as follows; Hs00153936_m1 for aggrecan (the core protein), Hs01028969_m1 for collagen type I, Hs00264051_m1 for collagen type II, Hs00181613_m1 for ANGPT1, Hs03986524_m1 for IGF-1, and Hs00218054_m1 for CSGALNACT1. NTP concentrations of 0.1 and 1.0 mNU/ml were used.

### Microarray

Gene expression in NP cells from four donors treated with or without NTP (1.0 mNU/ml) were compared using microarray analysis (N = 4). The addition of NTP was combined with 50 μg/ml AsAP. Total RNA was prepared as described above. Subsequently, Cy3-labeled cRNA was prepared from all four pairs using a Low Input Quick Amp Labeling Kit (#5190–2943, Agilent Technology, Santa Clara CA), and hybridized on a SurePrint G3 Human GE 8×60K v2 Microarray (#0306478915, Agilent Technology) using a Gene Expression Hybridization Kit (#5188–5242, Agilent Technology), and analyzed using an Agilent DNA Microarray Scanner (G2600D SG13164306) with the AgilentG3_HiSen_GX_1Color protocol (Agilent Technology). Probe level data were converted to expression values using the Agilent Feature Extraction 11.5.1.1 (Agilent Technology). Gene Spring ver.13 (Agilent Technology) was used for 75-percentile normalization, detection of genes with an average two-fold or higher change in expression, and for graphing. The cut-off point for normalized signal levels was set to minus five to exclude data with extremely low signals. The signals from multiple probes for the same gene were averaged and used as one data point. The Database for Annotation, Visualization and Integrated Discovery (DAVID) 2017 Tool (U.S. National Institutes of Health at https://david.ncifcrf.gov) and Kyoto Encyclopedia of Genes and Genomes (KEGG) PATHWAY Database (http://www.genome.jp/kegg) were used for gene ontology assessment and grouping of genes for the PI3K–AKT signaling pathway (has04151) and GAG synthetic groups (hsa00531, hsa00532, hsa00533) respectively.

### Western blotting

Expression of pAKT protein in the cells was investigated with addition of NTP alone or AsAP alone. At 30 and 60 minutes after the treatment with NTP (0.1,1.0 and 10 mNU/ml), NP cells were collected (N = 2). The cells treated with AsAP alone were collected after 60 minutes (N = 2). For detection of CSGALNACT1 protein we started cell cultures, in which cells received the combimed treatments with NTP (0.1 and 1.0 mNU/ml) and AsAP, and harvested them at one-week and two-week time points (N = 1). Cells were lysed in ice-cold cell lysis buffer (50 mM Tris-HCl, pH 7.5; #316–90221 and 1% Triton X-100; #595–13135, both from Wako Pure Chemical: 2 mM CaCl_2_; C1016, Sigma-Aldrich) containing protease and phosphatase inhibitors (0.5 mM phenylmethylsulfonylfluoride; #10837091001, Sigma-Aldrich:1/50 Complete, a protease inhibitor cocktail; #11873580001, Roche Molecular Biochemicals, Mannheim, Germany: 1 mM Na_3_VO_4_; #450243 and 1 mM NaF; S7920, Sigma-Aldrich). Protein concentrations were measured using a BCA protein assay Kit (#23225, Thermo Fisher Science). Equal amounts of protein (3 μg) were immunoblotted using the described antibodies. Concentrations of the primary antibodies were as follows, anti-AKT; 1:1000, anti-pAKT; 1:1000, anti-CSGALNACT1; 1:500, and anti-GAPDH;1:2000.

### Statistical analysis

Data are presented as average ± standard error of the mean (SEM). Statistical significance was assessed by repeated-measures analysis of variance and the Bonferroni correction for the results from cell proliferation assay. Differences between groups were analyzed by the Paired *t* test for the other experimental results.** Indicates highly significant differences (*p* < 0.01), * indicates significant differences (*p* < 0.05), when compared with control, ##*p* < 0.01, #*p* < 0.05 when compared with AsAP alone, and †† *p* < 0.01, when compared between NTP–treated samples, throughout the current study.

## Results and discussion

### Effect of NTP on cellular growth and GAG synthesis in NP cells

**Fig 1:**
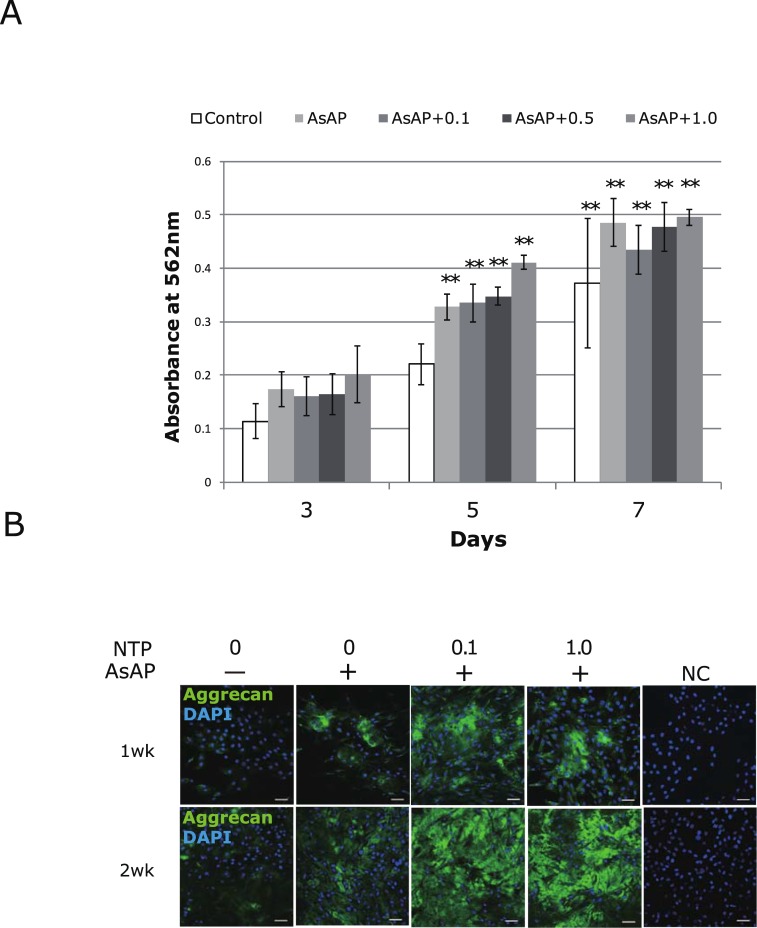
Effect of Neurotropin (NTP) on cellular growth and aggrecan synthesis. A) NP cells seeded in 96-well plates were cultured in 10% FBS α-MEM supplemented with 0, 0.1, 0.5, or 1.0 mNU/ml NTP with 50 μg/ml L-ascorbic acid-2-phosphate (AsAP). Cell proliferation was evaluated using the MTT assay on days 3, 5, and 7 after treatment, and values are shown as absorbance at 562 nm. Cell proliferation was enhanced on days 5 and 7 by either addition of AsAP or cotreatment with AsAP and NTP, compared with untreated cells on day 3. Data are presented as the average ± standard error (**p < 0.01, N = 3). B) Immunofluorescence staining of aggrecan in NTP-treated NP cells. Cells were seeded at a density of 3x10^3^ cells /cm^2^ on glass chamber slides and cultured with NTP. After two weeks of culture, expression of aggrecan core protein increased in the NTP-treated cultures compared with untreated cultures or those treated only with AsAP. Representative images are shown. Green indicates aggrecan, and nuclei are shown in blue by staining with 4′,6-diamidino-2-phenylindole dihydrochloride (DAPI). NTP concentrations of 0.1 and 1.0 mNU/ml were used. Bars are 50 μm.

**Fig 2:**
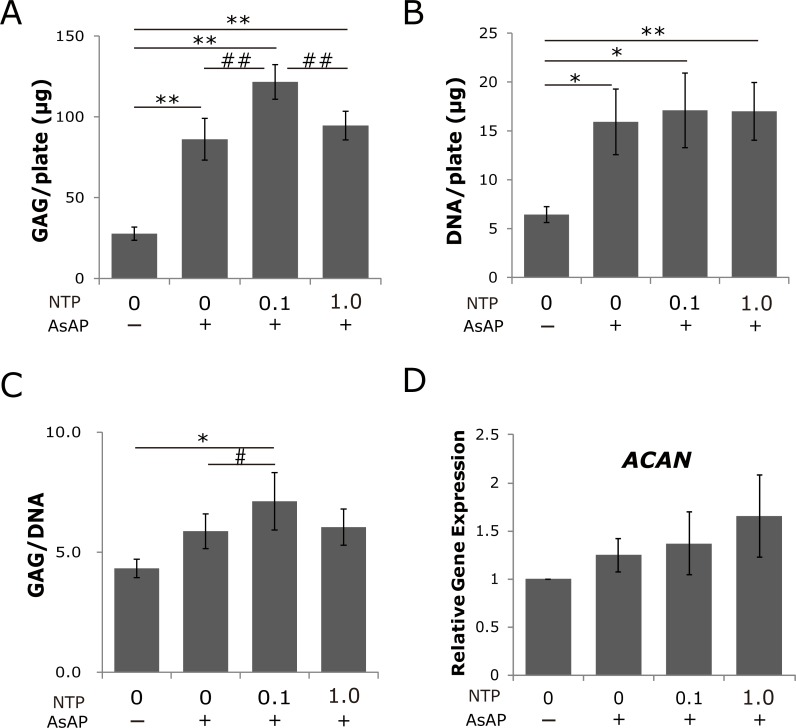
Effect of Neurotropin (NTP) on production of sulfated glycosaminoglycans. A) After two weeks of culture, glycosaminoglycan (GAG) content per plate increased in cultures with added AsAP, with or without the addition of NTP. B) DNA content increased in cultures with added AsAP, with or without the addition of NTP. C) GAG content normalized to DNA content was significantly upregulated by addition of 0.1 mNU/ml NTP with AsAP. D) After one week of culture, the effect of NTP on aggrecan (*ACAN*) mRNA expression was measured by real-time quantitative PCR. Gene expression of *ACAN* normalized to GAPDH showed a tendency to increase with increasing concentrations of NTP. Data are presented as the average ± standard error (**p < 0.01, *p < 0.05 when compared with control, ##*p* < 0.01, #*p* < 0.05 when compared with AsAP alone, and †† *p* < 0.01, when compared between NTP–treated samples, N = 5).

To assess the efficacy of the existing drug NTP in facilitating cellular GAG production, cultured human NP cells were treated with NTP. No negative influences on cellular proliferation were observed with NTP treatment in the range of 0.1 to 1.0 mNU/ml (Fig 1A, N = 3). The expression of aggrecan core protein as assessed by immunofluorescence microscopy increased after two weeks in the NTP-treated cultures, compared with the untreated cultures or those supplemented with AsAP alone (Fig 1B, N = 1). Quantification of sulfated GAG after two weeks of overconfluent cultures indicated that it was increased by treatment with AsAP (3.11 ± 0.47 times higher) or the combined addition of AsAP and NTP (0.1 or 1.0 mNU/ml) (4.39 ± 0.39 times higher or 3.42 ± 0.32 times higher, respectively) compared with control (all, *p* < 0.01; Fig 2A). Similarly, the DNA content was increased significantly by treatment with AsAP only (2.48 ± 0.52, *p* < 0.05) or combined with NTP (2.66 ± 0.59, *p* < 0.05, and 2.64 ± 0.46, *p* < 0.01; Fig 2B). However, the value of GAG normalized to DNA content was significantly upregulated only in the cultures treated with 0.1 mNU/ml NTP combined with AsAP (1.65 ± 0.28, *p* < 0.05; Fig 2C). Although the increase of the ascorbic acid concentration to 100 μg/ml (the basal medium contained 50 μg/ml ascorbic acid) significantly promoted DNA synthesis associated with cellular proliferation, this did not increase the GAG/DNA significantly (1.36 ± 0.17; Fig 2C). The combined treatment with higher concentration of AsAP with 1.0 mNU/ml NTP also had no significant effect compared with control (1.40 ± 0.17; Fig 2C). These results suggest that although GAG production in each cell (GAG/DNA) cannot be stimulated by addition of AsAP alone, it is stimulated by co-treatment with AsAP and 0.1 mNU/ml NTP. We then isolated the total RNA from the one-week cultures for quantitative analysis of mRNA encoding aggrecan (*ACAN*) core protein. As shown in Fig 2D, the level of *ACAN* mRNA tended to increase with increasing concentrations of NTP. This result was consistent with the images from immunofluorescence staining of aggrecan core protein (Fig 1B). Next, some of these total RNA samples were subjected to microarray analysis.

### Gene expression profiling

As our initial observations suggested that NTP had some stimulatory effect on cellular GAG production, we performed a microarray analysis to investigate differential gene expression between control NP cells and NP cells treated with 1.0 mNU/ml NTP combined with AsAP, which showed the highest level of *ACAN* mRNA. The reason why we chose these samples for microarray was because difference in mRNA expression levels of aggrecan between them was the largest (Fig 2D), although the value of GAG/DNA was the highest in the NP cells treated with 0.1 mNU/ml NTP combined with AsAP. Also, GAG was analyzed with two-week cultures, whereas, the total RNA samples for both microarray and qPCR were prepared from one-week cultures, so we made a choice based on the gene expression levels of aggrecan. Each group contained four biological replicates. The raw data for hybridization with the essential sample annotations including experimental factors and the final normalized data are shown in Supporting Information (S1 Appendix; https://www.ncbi.nlm.nih.gov/geo/, accession number GSE114169).

**Fig 3:**
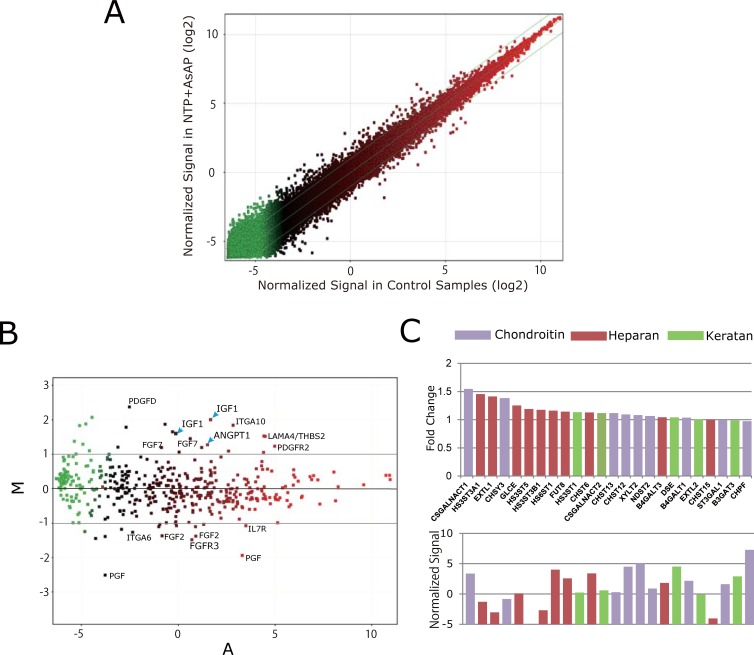
Gene expression profiling. A) Genes upregulated more than two-fold compared with control (above the upper green line), downregulated more than two-fold below control (below the lower green line), and genes that changed less than two-fold (between the green lines) are plotted logarithmically for 1.0 mNU/ml NTP with AsAP (1.0NTP+AsAp) treated samples versus control. Data were normalized for 75-percentile. B) MA plot for genes belonging to the phosphatidylinositol 3-kinase (PI3K) signaling pathway selected by KEGG PATHWAY Database. Genes upregulated or downregulated two-fold or more compared with control (above/below the green line), and genes with less than two-fold changes (between the green lines) are plotted. M = log_2_ (1.0NTP+AsAP) -log_2_ Control, A = [log_2_ (1.0NTP+AsAP) -log_2_ Control] / 2. C) Genes related to the sulfated glycosaminoglycan synthesis were analyzed and the top 24 genes by the magnitude of change resulting from 1.0NTP+AsAP treatment are shown (upper panel) with the differences in signal levels (lower panel). Chondroitin sulfate N-acetylgalactosaminyltransferase 1 (CS*GALNACT1*) was found to be upregulated 1.54 times compared with control. Data are presented as the average of N = 4.

The analysis indicated that 697 genes were upregulated more than two-fold by NTP treatment (Fig 3A). Functional analysis of the upregulated genes using DAVID identified diverse groups of genes in the category of biological processes (Table 1 and Table 3 in Supporting Information, *p* < 0.05). Within the lists, we focused on the positive regulation of PI3K signaling (*p* = 0.018) because this pathway has been known to be implicated in proteoglycan synthesis in cultured articular chondrocytes [30,31]. We then used KEGG pathway analysis to separate out the PI3K-related genes from all normalized data (Fig 3B). A number of genes, including *LAMA4*, *THBS2*, *PDGFR2*, *ITGA10*, *IGF1*, *ANGPT1*, *FGF7*, and *PDGFD*, were upregulated more than two-fold. However, *IL7R*, *PGF*, *FGF2*, *FGFR3*, and *ITGA6* were downregulated to less than 50% of control. Notably, elevated expression of *IGF1* (4.4 times) and angiopoietin 1 protein (*ANGPT1*,(2.8 times)) were found, both of which can facilitate signal transduction through PI3K via their specific receptors, IGF1 receptor and Tie2 receptor, respectively [35,36].

In the same way, genes related to sulfate GAG synthesis were identified, and the top 24 genes by the magnitude of change with NTP treatment are shown in Fig 3C, upper panel. Based on their functional annotations, they were classified into three groups: chondroitin sulfate (CS), heparan sulfate, and keratin sulfate groups. Although all changes were less than two-fold in magnitude, chondroitin sulfate N-acetylgalactosaminyltransferase 1 (*CSGALNACT1*) was found to be upregulated 1.54 times compared with control. Considering the magnitude of the differences in signal levels for expression of individual genes (Fig 3C, lower panel), *CSGALNACT1* showed the greatest change among the genes associated with sulfate GAG synthesis. These results suggest that *CSGALNACT1* may be a major effector of the upregulation of GAG production by NTP, which we measured using DMMB (Fig 2). In addition, genes associated with cellular responses to hypoxia were positively modulated by NTP treatment whereas genes associated with angiogenesis were negatively modulated (*p* = 6.4E-03 and *p* = 1.4E-02, Table 1). These changes might favor the maintenance of the specific characteristics of NP cells that reside in avascular conditions [37,38]. In addition to Table 1 a supplemental list for other groups of genes that were significantly upregulated by treatment with NTP is shown (P<0.05, S1 Table). There seemed to be no biological process associated with cytotoxicity or oncogenic effect.

**Table 1:** Functional analysis of the 697 genes upregulated more than two-fold by NTP using DAVID vir6.8 functional annotation tool.

**Term**	**Count**	***p*-value**^**a**^
**GO:0007275 multicellular organism development**	41	1.3E–04
**GO:0007166 cell surface receptor signaling pathway**	23	2.4E–03
**GO:0055085 transmembrane transport**	21	3.0E–03
**GO:0001523 retinoid metabolic process**	9	3.5E–03
**GO:0007165 signal transduction**	67	5.5E–03
**GO:0071456 cellular response to hypoxia**	11	6.4E–03
**GO:0043547 positive regulation of GTPase activity**	37	7.1E–03
**GO:0071773 cellular response to BMP stimulus**	6	7.3E–03
**GO:0007155 cell adhesion**	31	9.4E–03
**GO:0016338 calcium-independent cell–cell adhesion via plasma membrane cell-adhesion molecules**	5	1.0E–02
**GO:0034394 protein localization to cell surface**	5	1.2E–02
**GO:0030282 bone mineralization**	6	1.2E–02
**GO:0010628 positive regulation of gene expression**	20	1.3E–02
**GO:0016525 negative regulation of angiogenesis**	8	1.4E–02
**GO:0030177 positive regulation of Wnt signaling pathway**	6	1.6E–02
**GO:0001819 positive regulation of cytokine production**	5	1.6E–02
**GO:0014068 positive regulation of phosphatidylinositol 3-kinase signaling**	8	1.8E–02
**GO:0030335 positive regulation of cell migration**	15	2.1E–02
**GO:1901215 negative regulation of neuron death**	6	2.4E–02
**GO:0008285 negative regulation of cell proliferation**	26	2.5E–02
**GO:0051496 positive regulation of stress fiber assembly**	6	2.9E–02
**GO:0070374 positive regulation of ERK1 and ERK2 cascade**	14	3.0E–02
**GO:2000427 positive regulation of apoptotic cell clearance**	3	3.1E–02
**GO:0045785 positive regulation of cell adhesion**	6	3.2E–02
**GO:0030198 extracellular matrix organization**	15	3.4E–02
**GO:0044344 cellular response to fibroblast growth factor stimulus**	5	3.4E–02
**GO:0007267 cell–cell signaling**	18	3.6E–02
**GO:0045669 positive regulation of osteoblast differentiation**	7	3.7E–02
**GO:0032332 positive regulation of chondrocyte differentiation**	4	4.2E–02
**GO:0035115 embryonic forelimb morphogenesis**	5	4.2E–02
**GO:0045600 positive regulation of fat cell differentiation**	6	4.4E–02

^a^Major biological processes are ordered according to their attributed descriptive *p*-value.

### NTP activates AKT phosphorylation

**Fig 4:**
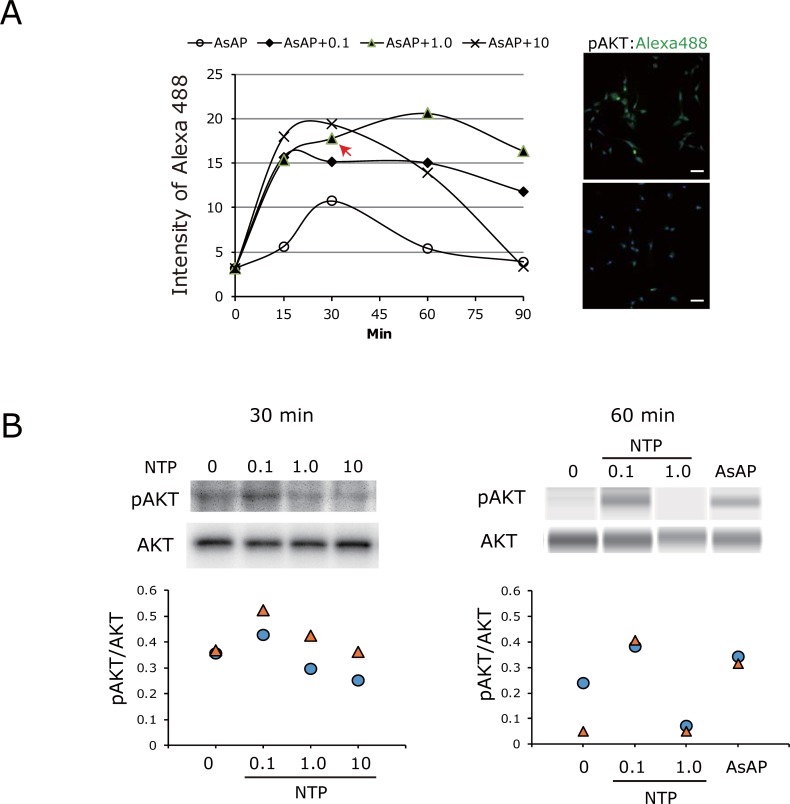
NTP activates AKT phosphorylation. Phosphorylation of AKT by NTP was examined in NP cells by staining with specific antibody against pAKT (Ser473). A) Time courses show the integrated signals of the Alexa Fluor 488-labeled pAKT (shown in green in the photograph) indicated peaks from 15 to 90 minutes after addition of NTP and AsAP. The peak levels of phosphorylation were much higher in the samples co-treated with 0.1, 1.0 and 10 mNU/ml NTP and AsAP than in those treated with AsAP only. Capture of immunofluorescence images and integration of the signal intensity in the cultured cells were performed using the ArrayScan system. Every serial sample was assayed in duplicated wells (Donor, N = 1). Red arrow shows the time point at which the representative photographs were taken. Nuclei are shown in blue by staining with DAPI. Bars are 100 μm. B) Western blot analysis of phosphorylation of AKT using the same antibody. Thirty minutes and 60 minutes after addition of NTP, cells were harvested and whole cell lysate was immunoblotted (N = 2). Addition of 0.1 mNU/ml NTP upregulated phosphorylation of AKT (Ser473) compared with control, but higher concentrations of NTP (1.0 and 10 mNU/ml) were not proportionally more effective (left and right). Addition of AsAP alone was also effective on AKT phosphorylation (right). AKT is constant under all culture conditions.

To evaluate downstream signaling evoked by NTP, the phosphorylation of AKT was examined in NP cells by staining with specific antibody against pAKT (Ser473). Capture of images and integration of the fluorescence intensity in the cultured attached cells were performed using an ArrayScan system. The time courses indicated that the integrated signal from the Alexa Fluor 488-labeled pAKT showed peaks from 15 to 90 minutes after addition of NTP and AsAP. The peak levels of phosphorylation were much higher in the samples co-treatmed with 0.1,1.0, and 10 mNU/ml NTP and AsAP than in those treated with AsAP only (Fig 4A). Next, to confirm effect of NTP itself cells were treated with NTP without AsAp, and whole cell lysate was analyzed by western blotting using the same antibody. The results indicate that NTP (0.1 mNU/ml) treatment upregulated phosphorylation of AKT (Ser473) compared with control; however, higher concentrations of NTP (1.0 and 10 mNU/ml) were not proportionally more effective. Note that addition of AsAP alone was also effective on AKT phosphorylation (Fig 4B). Further study is needed to define the optimum dose of NTP for addition to culture medium and for *in vivo* applications.

### Upregulation of *IGF1*, *ANG1*, and *CSGALNACT1* expression by NTP

**Fig 5:**
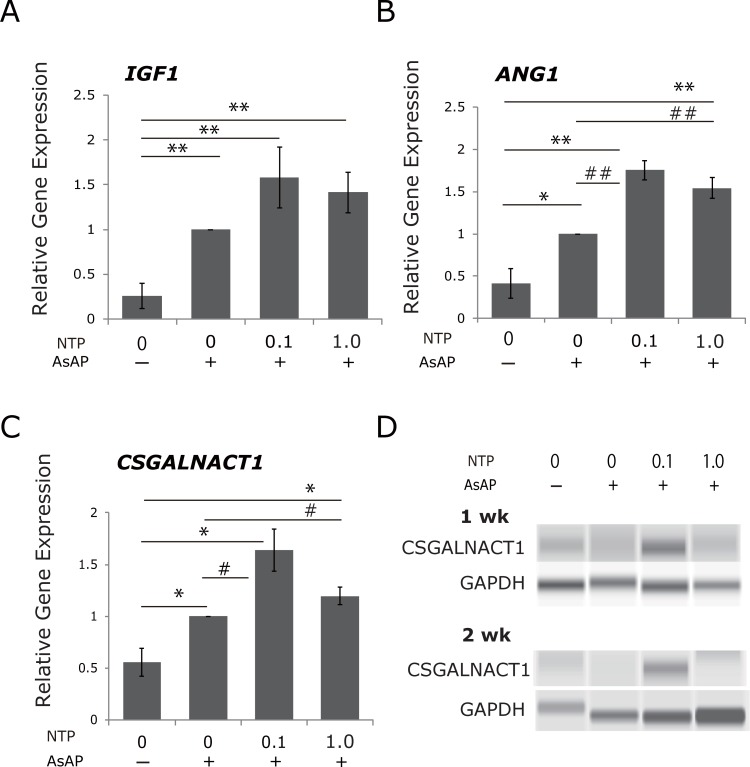
Upregulation of *IGF1*, *ANG1*, and *CSGALNACT1* expression by NTP treatment. Based on the results of the microarray, relative mRNA expression of *IGF1*, *ANG1*, and *CSGALNACT1* was assessed by real-time quantitative PCR. Samples were prepared form one-week-cultured cells, treated as indicated. A-C) Addition of AsAP with or without NTP upregulated expression of these three genes in comparison with control samples. A) The co-treatment with NTP (0.1 or 1.0 mNU/ml) and AsAP tended to increase *IGF1* compared to AsAP alone. B) The co-treatment with NTP (0.1 or 1.0 mNU/ml) and AsAP increased *ANG1* significantly compared to AsAP alone. C) The co-treatment with NTP (0.1 mNU/ml) and AsAP increased *CSGALNACT1* significantly compared to AsAP alone. Data are normalized to GAPDH and presented as the average ± standard error (**p < 0.01, *p < 0.05 when compared with control, ##*p* < 0.01, #*p* < 0.05 when compared with AsAP alone, N = 5). D) Whole cell lysate prepared from one-week and two-week-cultured NP cells was analyzed by western blotting with an appropriate antibody (N = 1). Enhanced expression of CSGALNACT1 by the co-treatment with 0.1 mNU/ml NTP and AsAP in protein levels at one-week and two-week time points was detected. Staining for GAPDH was shown as loading control.

The most striking evidence to come from the gene expression profiling was the elevated expression of *IGF1*, *ANG1* (for *ANGPT1* in short henceforth), and *CSGALNACT1* in the NTP-treated group. We confirmed expression of these three genes by real-time PCR. As results, addition of AsAP either with or without NTP upregulated expression of these genes in comparison to control samples (all, *p* < 0.01 or *p* < 0.05, Fig 5A–5C). Therefore, we showed the ratio of the gene expression level in each sample to the level in the sample treated with AsAP alone. Obviously, the highest expression in all of these genes is induced by the co-treatment with 0.1 mNU/ml NTP and AsAP. In comparison to AsAP alone, the co-treatments with NTP (0.1 or 1.0 mNU/ml) tended to increase *IGF1* (1.58± 0.34 times higher, and 1.41 ± 0.23 times higher, respectively, Fig 5A). The co-treatments with NTP (0.1 or 1.0 mNU/ml) and AsAP showed significant differences in *ANG1* expression; upregulations (1.75 ± 0.11 times, 1.54 ± 0.27 times) compared to AsAP alone (both *p* < 0.01; Fig 5B). In the case of *CSGALNACT*, the co-treatments with NTP (0.1 or 1.0 mNU/ml) and AsAP also upregulated gene expression by 1.64 ± 0.21, 1.20 ± 0.08 times, compared to AsAP alone (both, *p* < 0.05; Fig 5C). Additionally, we detected enhanced expression of CSGALNACT1 by the co-treatment with 0.1 mNU/ml NTP and AsAP in protein levels in the samples prepared from one-week and two-week-cultured cells (Fig 5D). These results are consistent with the elevation of GAG production by the co-treatment (Fig 2C). Therefore, the results from real-time PCR and western blotting for CSGALNACT1 support the finding that addition of 0.1 mNU/ml NTP with 50 μg/ml AsAP may be optimum to stimulate GAG synthesis in cultured human NP cells. Surprisingly, the optimum dose setting for NTP found in the experimental results is very close to the concentration; 0.12 mNU/ml in blood plasma attained by an intravenous injection.

We previously reported the crucial roles of ANG1 in prevention of apoptosis in NP cells induced by serum withdrawal, and in maintenance of NP progenitor cells with evidence of expression of Tie2, a functional receptor for ANG1 in human and murine NP cells [11]. Similarly, other investigators have reported its protective effect against serum deprivation and hypoxia-induced apoptosis in mesenchymal stem cells, via the PI3K–AKT pathway [36]. The important role of IGF1 in proteoglycan synthesis in IVD cells has also been reported in previous studies [39–41].

### Contribution of ascorbic acid

Ascorbic acid has been reported to be essential for the synthesis of collagen and also acts as an antioxidant in the IVD [42]. Previous investigators reported that it works as a cofactor for the hydroxylation of lysine and proline, which form the intermolecular bonds of the collagen triple helix in cartilage. In the presence of ascorbic acid, a larger proportion of the newly synthesized sulfated proteoglycans was deposited in the cell layer *in vitro* [43]. In this study, NP cells grew and reached confluency much faster in the presence of ascorbic acid, which might have facilitated accumulation of GAG in the later days of the two-week experimental period. In the microarray analysis we did not include the samples treated with AsAP alone, which had not shown obvious difference in mRNA expression of aggrecan (Fig 2D). However, AsAP itself, a stable analog of ascorbic acid cannot be disregarded for future applications of NP cells either *in vitro* or *in vivo* setting, because of its effects on phosphorylation of AKT and enhancement in gene expression (Figs 1,2,4 and 5).

### Synthesis of chondroitin sulfate

The largest and most important proteoglycan in the disc matrix is aggrecan, which is made up of a core protein with negatively charged GAG side chains composed of CS and keratin sulfate, which are capable of absorbing water [27,44]. In the central region of the NP, the aggrecan molecules accumulate on a hyaluronan strand to form structures called aggregate, in which the abundant CS provides osmotic properties, including the ability to swell and resist compressive loads [44]. In this study, we detected elevated expression of the gene *CSGALNACT1*, which encodes an enzyme that initiates synthesis of CS polysaccharide chains by its activity as an N-acetylgalactosaminyltransferase 1 (CSGalNAcT-1) toward a tetrasaccharide of the GAG linkage structure (GlcA-Gal-Gal-Xyl-Ser) that links the CS-GAG chains onto the core protein of aggrecan. Another enzyme, CSGalNAcT-2, which is highly homologous to CSGalNAcT-1, functions to elongate the poly- and oligosaccharide chains with a β 1–4 linkage [45]. In this study, we detected a slight increase in expression of the gene for CSGalNAcT-2 after NTP treatment (1.12 times), but this change was much smaller than that of CSGalNAcT-1 (1.54 times increase; Fig 3C). These results suggest that treatment with NTP preferentially increases the number of CS-GAG chains rather than elongating them. Turnover of aggrecan in adult human IVDs has been reported to decrease over a lifetime [7,10]. The mean turnover rate for normal aggrecan is 0.126 per year and the mean half-life (defined as ln(2)/turnover rate) is 5.7 years, which increases with aging. In contrast to the effect of aging, turnover is consistently higher for degenerate IVDs than for normal IVDs at the same age. Moreover, aggrecan does not maintain a constant structure and abundance throughout life, because it has little inherent resistance to proteolytic degradation. Most proteinases that have access to aggrecan will cleave it in one or more of its domains [7]. In this study, NTP treatment showed a tendency to increase levels of mRNA for the aggrecan core proteins and significant increase in GAG production with elevated expression of mRNA and protein for CSGalNAcT-1. All these results suggest that NTP treatment could facilitate an increase in the number of CS-GAG chains and restore the ability of aggrecan molecules to absorb water, thereby encouraging the regeneration of NP cells from herniated IVDs.

## Conclusions

In this study, we demonstrated a novel anabolic effect of NTP in stimulating proteoglycan production by human NP cells isolated from patients with herniated lumbar discs. Thus, NTP may be clinically useful for enhancing the synthesis of CS-GAG in the IVD to attenuate or reverse the degenerative process, because stimulation of CS-GAG production in the damaged NP tissue may help recover the nuclear swelling pressure and prevent the loss of IVD structural integrity. Furthermore, a high concentration of aggrecan molecules are an impediment to blood vessel [46] and nerve ingrowth into IVD tissue [28]. These effects could be helpful to prevent damaged IVD from the onset of inflammation and sensitization, both of which associate with pain. As shown by our results, NTP upregulates phosphorylation of AKT and downstream signal propagation evoked by NTP influences expression of a large and diverse group of genes to increase *IGF1* and *ANG1* expression in an autocrine manner and leads to upregulation of GAG synthesis by CSGalNAcT-1. Therefore, NTP may function both directly and indirectly to stimulate GAG production in NP cells. Moreover, the rarity of side effects of NTP is a major advantage for its clinical application. It may also be possible to use ascorbic acid together with NTP, because it is clinically available and uptake by NP cells has already been reported [42]. However, the avascular environment of the IVD remains an issue that must be resolved. To address this, methods for delivering appropriate concentrations of NTP into the IVD locally or systemically need to be devised using animal models. Overall, NTP treatment provides a possible way to ameliorate clinical outcomes of LBP by stimulating GAG production within damaged IVD tissues, where biological and mechanical resilience is threatened.

## Supporting information

S1 Appendix Raw data for each hybridization. (PDF)

S1 Table Complement of the functional analysis of the 697 genes upregulated higher than two-fold by NTP: List of biological processes implicated in response to extraneous material. (PDF)
