# Supplementary material for: Upregulation of glycosaminoglycan synthesis by Neurotropin in nucleus pulposus cells via stimulation of chondroitin sulfate N-acetylgalactosaminyltransferase 1: A new approach to attenuation of intervertebral disc degeneration
Source: PLoS One. 2018 Aug 27;13(8):e0202640. doi: 10.1371/journal.pone.0202640 (PMC6110471; doi:10.1371/journal.pone.0202640)
Supplement: S1 Appendix — (PDF) [file pone.0202640.s001.pdf]

Supporting Information 1

Sample annotation in the raw data for maicroarray analysis

slide position	Donor	Sample	ProcessedSignal(normalized)
1-1	1	Control_1	SG13164306_253949447586_S001_GE1_1105_Oct12_1_1
1-2	1	NTP+As.AP_1	SG13164306_253949447586_S001_GE1_1105_Oct12_1_2
1-3	2	Control_2	SG13164306_253949447586_S001_GE1_1105_Oct12_1_3
1-4	2	NTP+As.AP_2	SG13164306_253949447586_S001_GE1_1105_Oct12_1_4
2-1	3	Control_3	SG13164306_253949447586_S001_GE1_1105_Oct12_2_1
2-2	3	NTP+As.AP_3	SG13164306_253949447586_S001_GE1_1105_Oct12_2_2
2-3	4	Control_4	SG13164306_253949447586_S001_GE1_1105_Oct12_2_3
2-4	4	NTP+As.AP_4	SG13164306_253949447586_S001_GE1_1105_Oct12_2_4

8 x 60K

1_1	1_2	1_3	1_4
2_1	2_2	2_3	2_4

QC Report - Agilent Technologies : 1 Color Gene Expression

Date	Tuesday, October 18, 2016 - 10:52	Grid	039494_D_F_20150612
Image	SG13164306_253949447586_S001 [2_3]	BG Method	No Background
Protocol	GE1_1105_Oct12 (Read Only)	Background Detrend	On(FeatNCRange, LoPass)
User Name	admin	Multiplicative Detrend	True
FE Version	11.5.1.1	Additive Error	2(Green)
Sample(red/green)		Saturation Value	776001 (g)

Spot Finding of the Four Corners of the Array

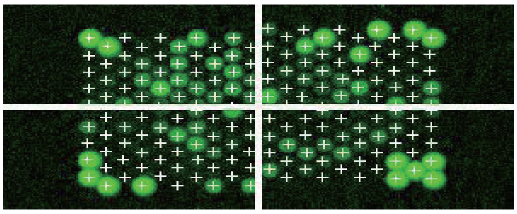

Grid Normal

	Feature Green	Local Background Green
Non Uniform	9	0
Population	622	1409

Spatial Distribution of All Outliers on the Array

384 rows x 164 columns

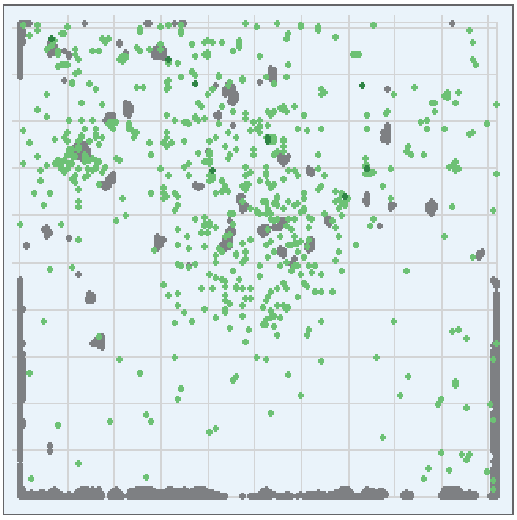

FeatureNonUnif (Green) = 9(0.01%)

GeneNonUnif (Green) = 8 (0.016 %)

- BG NonUniform
- BG Population
- Green FeaturePopulation
- Green Feature NonUniform

Negative Control Stats

	Green
Average Net Signals	13.88
StdDev Net Signals	2.36
Average BG Sub Signal	-1.97
StdDev BG Sub Signal	1.51

Net Signal Statistics

Agilent SpikeIns:

	Green
# Saturated Features	0
99% of Sig. Distrib.	203990
50% of Sig. Distrib.	480
1% of Sig. Distrib.	14

Non-Control probes:

	Green
# Saturated Features	0
99% of Sig. Distrib.	17109
50% of Sig. Distrib.	31
1% of Sig. Distrib.	12

Histogram of Signals Plot

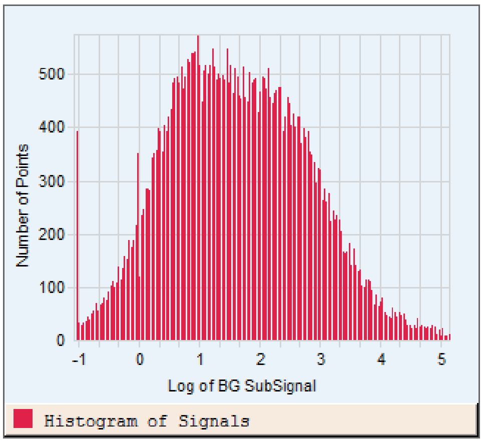

Features (NonCtrl) with BGSubSignal < 0: 10536 (Green)

QC Report - Agilent Technologies : 1 Color Gene Expression

Date	Tuesday, October 18, 2016 - 10:52	Grid	039494_D_F_20150612
Image	SG13164306_253949447586_S001 [1_4]	BG Method	No Background
Protocol	GE1_1105_Oct12 (Read Only)	Background Detrend	On(FeatNCRange, LoPass)
User Name	admin	Multiplicative Detrend	True
FE Version	11.5.1.1	Additive Error	1(Green)
Sample(red/green)		Saturation Value	776001 (g)

Spot Finding of the Four Corners of the Array

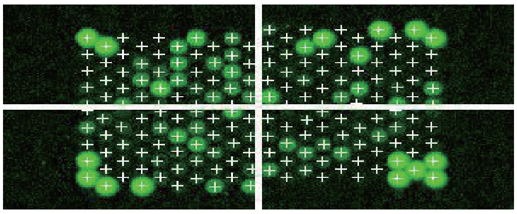

Grid Normal

	Feature Green	Local Background Green
Non Uniform	6	0
Population	443	1158

Spatial Distribution of All Outliers on the Array

384 rows x 164 columns

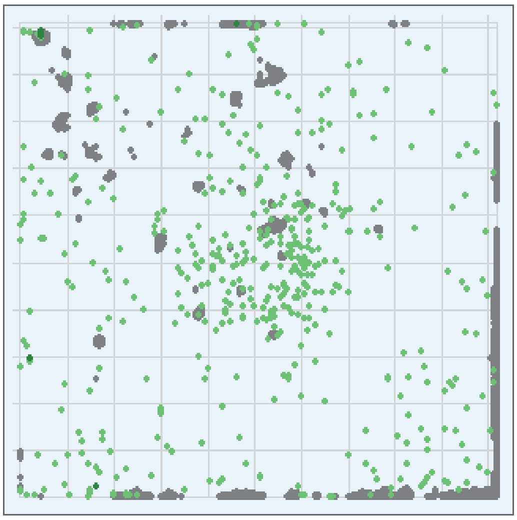

FeatureNonUnif (Green) = 6(0.01%)

GeneNonUnif (Green) = 5 (0.010 %)

- BG NonUniform
- BG Population
- Green FeaturePopulation
- Green Feature NonUniform

Negative Control Stats

	Green
Average Net Signals	13.70
StdDev Net Signals	1.25
Average BG Sub Signal	-1.29
StdDev BG Sub Signal	1.09

Net Signal Statistics

Agilent SpikeIns:

	Green
# Saturated Features	0
99% of Sig. Distrib.	217939
50% of Sig. Distrib.	596
1% of Sig. Distrib.	14

Non-Control probes:

	Green
# Saturated Features	0
99% of Sig. Distrib.	17109
50% of Sig. Distrib.	27
1% of Sig. Distrib.	12

Histogram of Signals Plot

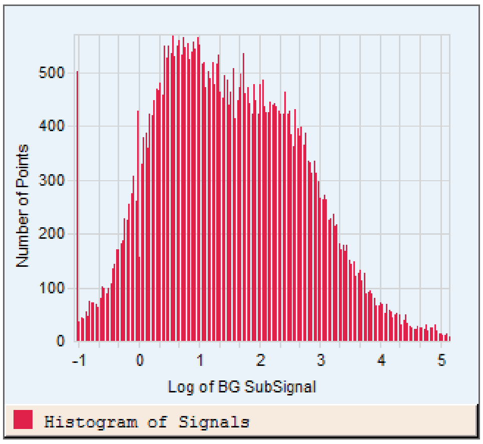

Features (NonCtrl) with BGSubSignal < 0: 8610 (Green)

QC Report - Agilent Technologies : 1 Color Gene Expression

Date	Tuesday, October 18, 2016 - 10:52	Grid	039494_D_F_20150612
Image	SG13164306_253949447586_S001 [1_3]	BG Method	No Background
Protocol	GE1_1105_Oct12 (Read Only)	Background Detrend	On(FeatNCRange, LoPass)
User Name	admin	Multiplicative Detrend	True
FE Version	11.5.1.1	Additive Error	1(Green)
Sample(red/green)		Saturation Value	776001 (g)

Spot Finding of the Four Corners of the Array

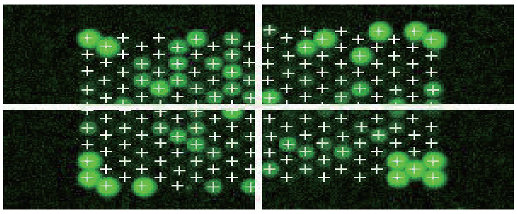

Grid Normal

	Feature Green	Local Background Green
Non Uniform	1	0
Population	411	2180

Spatial Distribution of All Outliers on the Array

384 rows x 164 columns

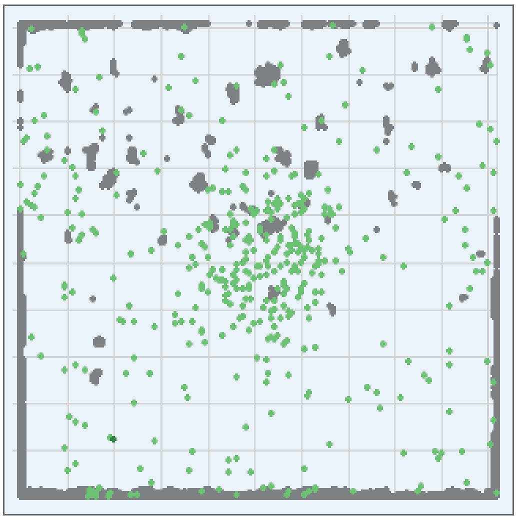

FeatureNonUnif (Green) = 1(0.00%)

GeneNonUnif (Green) = 1 (0.002 %)

- BG NonUniform
- BG Population
- Green FeaturePopulation
- Green Feature NonUniform

Negative Control Stats

	Green
Average Net Signals	13.81
StdDev Net Signals	1.41
Average BG Sub Signal	-1.45
StdDev BG Sub Signal	1.15

Net Signal Statistics

Agilent SpikeIns:

	Green
# Saturated Features	0
99% of Sig. Distrib.	212503
50% of Sig. Distrib.	523
1% of Sig. Distrib.	15

Non-Control probes:

	Green
# Saturated Features	0
99% of Sig. Distrib.	22577
50% of Sig. Distrib.	30
1% of Sig. Distrib.	12

Histogram of Signals Plot

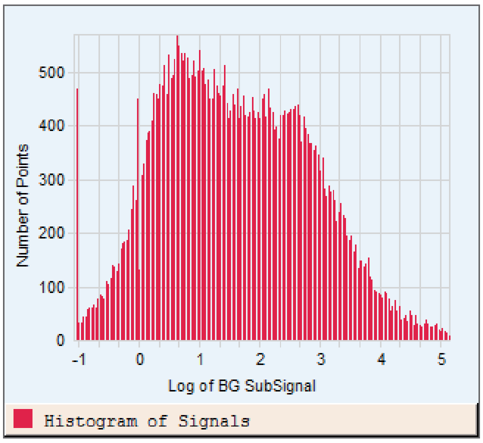

Features (NonCtrl) with BGSubSignal < 0: 8612 (Green)

QC Report - Agilent Technologies : 1 Color Gene Expression

Date	Tuesday, October 18, 2016 - 10:52	Grid	039494_D_F_20150612
Image	SG13164306_253949447586_S001 [1_2]	BG Method	No Background
Protocol	GE1_1105_Oct12 (Read Only)	Background Detrend	On(FeatNCRange, LoPass)
User Name	admin	Multiplicative Detrend	True
FE Version	11.5.1.1	Additive Error	1(Green)
Sample(red/green)		Saturation Value	776001 (g)

Spot Finding of the Four Corners of the Array

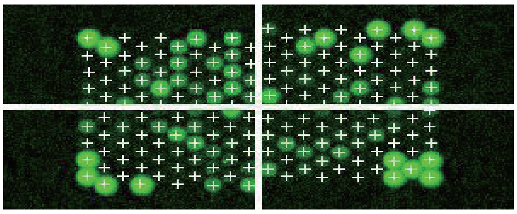

Grid Normal

	Feature Green	Local Background Green
Non Uniform	0	0
Population	416	2668

Spatial Distribution of All Outliers on the Array

384 rows x 164 columns

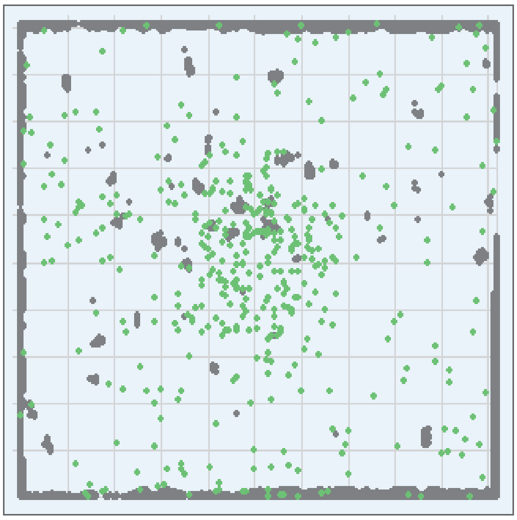

FeatureNonUnif (Green) = 0(0.00%)

GeneNonUnif (Green) = 0 (0.000 %)

•BG NonUniform
•Green FeaturePopulation
•BG Population
•Green Feature NonUniform

Negative Control Stats

	Green
Average Net Signals	13.59
StdDev Net Signals	1.37
Average BG Sub Signal	-1.30
StdDev BG Sub Signal	1.06

Net Signal Statistics

Agilent SpikeIns:

	Green
# Saturated Features	0
99% of Sig. Distrib.	223607
50% of Sig. Distrib.	585
1% of Sig. Distrib.	14

Non-Control probes:

	Green
# Saturated Features	0
99% of Sig. Distrib.	18778
50% of Sig. Distrib.	28
1% of Sig. Distrib.	12

Histogram of Signals Plot

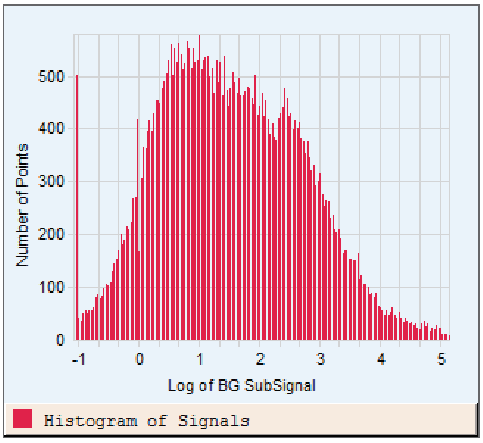

Features (NonCtrl) with BGSubSignal < 0: 8657 (Green)

QC Report - Agilent Technologies : 1 Color Gene Expression

Date	Tuesday, October 18, 2016 - 10:52	Grid	039494_D_F_20150612
Image	SG13164306_253949447586_S001 [1_1]	BG Method	No Background
Protocol	GE1_1105_Oct12 (Read Only)	Background Detrend	On(FeatNCRange, LoPass)
User Name	admin	Multiplicative Detrend	True
FE Version	11.5.1.1	Additive Error	1(Green)
Sample(red/green)		Saturation Value	776001 (g)

Spot Finding of the Four Corners of the Array

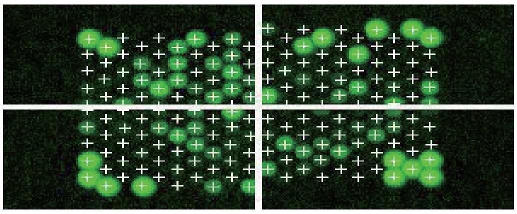

Grid Normal

	Feature Green	Local Background Green
Non Uniform	4	4
Population	457	4109

Spatial Distribution of All Outliers on the Array

384 rows x 164 columns

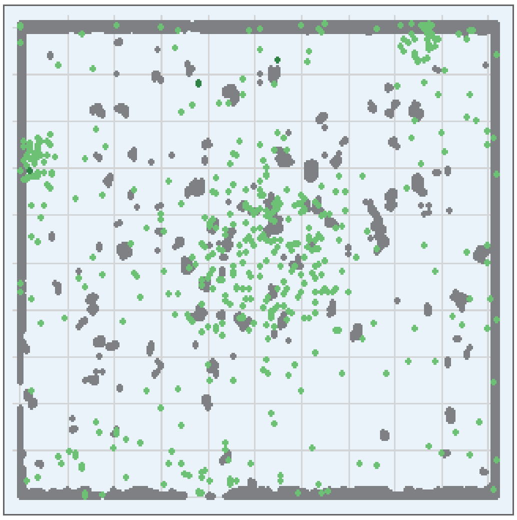

FeatureNonUnif (Green) = 4(0.01%)

GeneNonUnif (Green) = 4 (0.008 %)

- BG NonUniform
- BG Population
- Green FeaturePopulation
- Green Feature NonUniform

Negative Control Stats

	Green
Average Net Signals	13.58
StdDev Net Signals	1.20
Average BG Sub Signal	-1.14
StdDev BG Sub Signal	1.07

Net Signal Statistics

Agilent SpikeIns:

	Green
# Saturated Features	0
99% of Sig. Distrib.	220588
50% of Sig. Distrib.	558
1% of Sig. Distrib.	15

Non-Control probes:

	Green
# Saturated Features	0
99% of Sig. Distrib.	24350
50% of Sig. Distrib.	31
1% of Sig. Distrib.	12

Histogram of Signals Plot

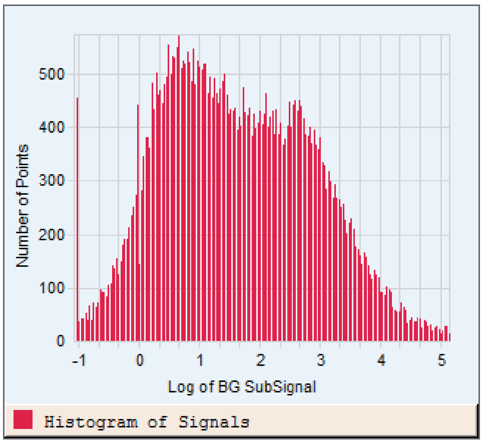

Features (NonCtrl) with BGSubSignal < 0: 7844 (Green)

QC Report - Agilent Technologies : 1 Color Gene Expression

Date	Tuesday, October 18, 2016 - 10:52	Grid	039494_D_F_20150612
Image	SG13164306_253949447586_S001 [2_2]	BG Method	No Background
Protocol	GE1_1105_Oct12 (Read Only)	Background Detrend	On(FeatNCRange, LoPass)
User Name	admin	Multiplicative Detrend	True
FE Version	11.5.1.1	Additive Error	2(Green)
Sample(red/green)		Saturation Value	776001 (g)

Spot Finding of the Four Corners of the Array

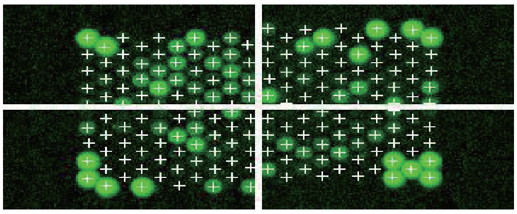

Grid Normal

	Feature Green	Local Background Green
Non Uniform	3	0
Population	575	1961

Spatial Distribution of All Outliers on the Array

384 rows x 164 columns

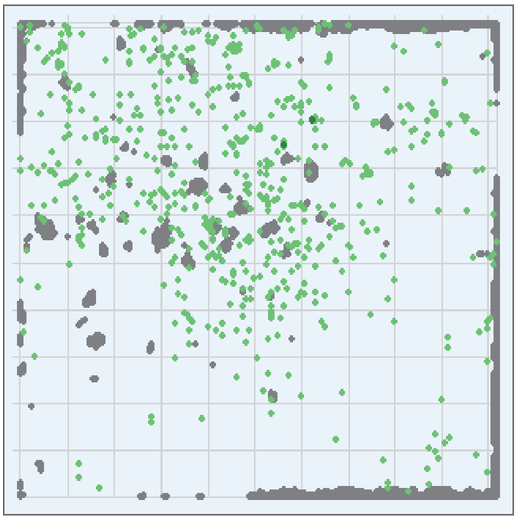

FeatureNonUnif (Green) = 3(0.00%)

GeneNonUnif (Green) = 2 (0.004 %)

•BG NonUniform •BG Population
•Green FeaturePopulation •Green Feature NonUniform

Negative Control Stats

	Green
Average Net Signals	13.58
StdDev Net Signals	2.12
Average BG Sub Signal	-1.94
StdDev BG Sub Signal	1.44

Net Signal Statistics

Agilent SpikeIns:

	Green
# Saturated Features	0
99% of Sig. Distrib.	225794
50% of Sig. Distrib.	743
1% of Sig. Distrib.	13

Non-Control probes:

	Green
# Saturated Features	0
99% of Sig. Distrib.	16575
50% of Sig. Distrib.	31
1% of Sig. Distrib.	11

Histogram of Signals Plot

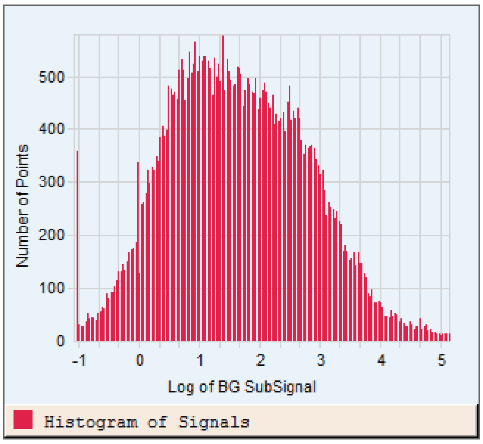

Features (NonCtrl) with BGSubSignal < 0: 10531 (Green)

QC Report - Agilent Technologies : 1 Color Gene Expression

Date	Tuesday, October 18, 2016 - 10:52	Grid	039494_D_F_20150612
Image	SG13164306_253949447586_S001 [2_1]	BG Method	No Background
Protocol	GE1_1105_Oct12 (Read Only)	Background Detrend	On(FeatNCRange, LoPass)
User Name	admin	Multiplicative Detrend	True
FE Version	11.5.1.1	Additive Error	2(Green)
Sample(red/green)		Saturation Value	776001 (g)

Spot Finding of the Four Corners of the Array

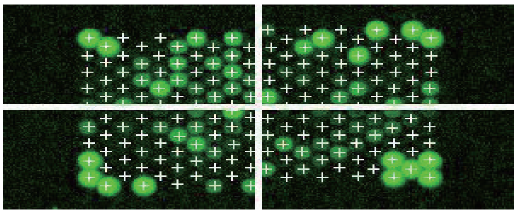

Grid Normal

	Feature Green	Local Background Green
Non Uniform	7	0
Population	591	3858

Spatial Distribution of All Outliers on the Array

384 rows x 164 columns

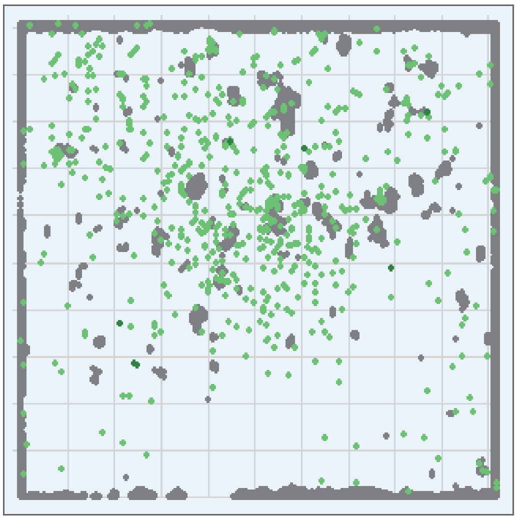

FeatureNonUnif (Green) = 7(0.01%)

GeneNonUnif (Green) = 7 (0.014 %)

- BG NonUniform
- BG Population
- Green FeaturePopulation
- Green Feature NonUniform

Negative Control Stats

	Green
Average Net Signals	13.89
StdDev Net Signals	2.06
Average BG Sub Signal	-2.04
StdDev BG Sub Signal	1.46

Net Signal Statistics

Agilent SpikeIns:

	Green
# Saturated Features	0
99% of Sig. Distrib.	210442
50% of Sig. Distrib.	444
1% of Sig. Distrib.	14

Non-Control probes:

	Green
# Saturated Features	0
99% of Sig. Distrib.	21196
50% of Sig. Distrib.	32
1% of Sig. Distrib.	12

Histogram of Signals Plot

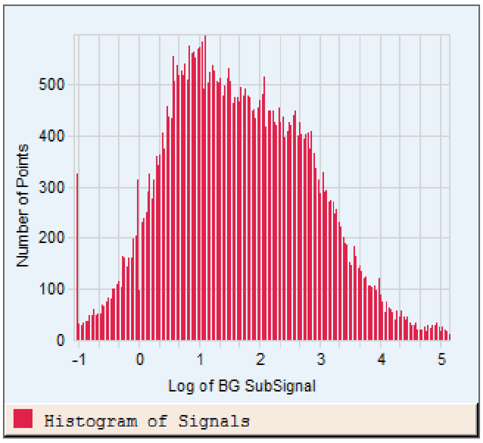

Features (NonCtrl) with BGSubSignal < 0: 9760 (Green)

QC Report - Agilent Technologies : 1 Color Gene Expression

Date	Tuesday, October 18, 2016 - 10:52	Grid	039494_D_F_20150612
Image	SG13164306_253949447586_S001 [2_4]	BG Method	No Background
Protocol	GE1_1105_Oct12 (Read Only)	Background Detrend	On(FeatNCRange, LoPass)
User Name	admin	Multiplicative Detrend	True
FE Version	11.5.1.1	Additive Error	2(Green)
Sample(red/green)		Saturation Value	776001 (g)

Spot Finding of the Four Corners of the Array

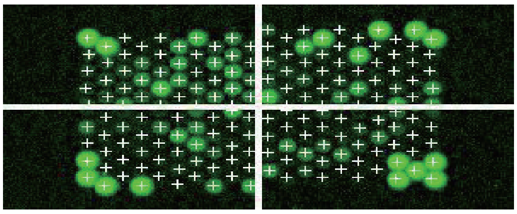

Grid Normal

	Feature Green	Local Background Green
Non Uniform	1	0
Population	601	1002

Spatial Distribution of All Outliers on the Array

384 rows x 164 columns

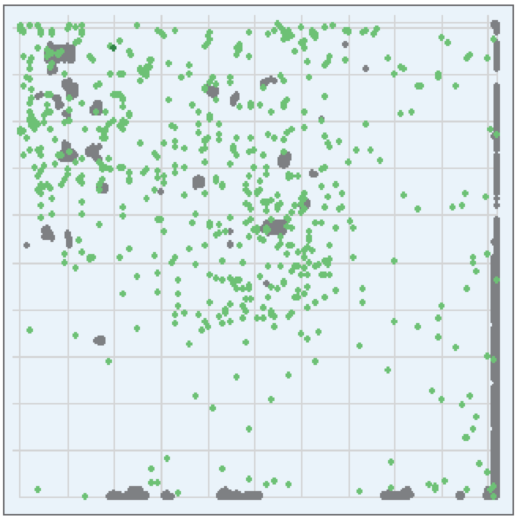

FeatureNonUnif (Green) = 1(0.00%)

GeneNonUnif (Green) = 0 (0.000 %)

- BG NonUniform
- BG Population
- Green FeaturePopulation
- Green Feature NonUniform

Negative Control Stats

	Green
Average Net Signals	13.61
StdDev Net Signals	1.97
Average BG Sub Signal	-1.59
StdDev BG Sub Signal	1.31

Net Signal Statistics

Agilent SpikeIns:

	Green
# Saturated Features	0
99% of Sig. Distrib.	199605
50% of Sig. Distrib.	504
1% of Sig. Distrib.	13

Non-Control probes:

	Green
# Saturated Features	0
99% of Sig. Distrib.	15182
50% of Sig. Distrib.	29
1% of Sig. Distrib.	11

Histogram of Signals Plot

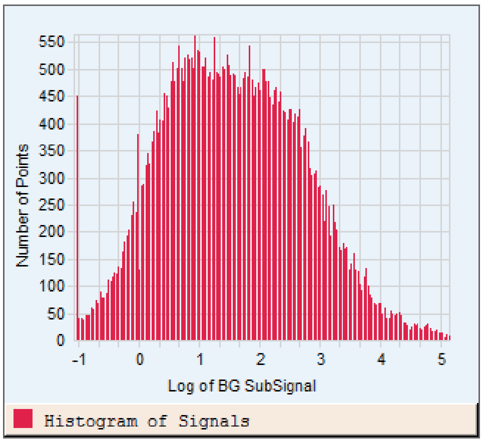

Features (NonCtrl) with BGSubSignal < 0: 10196 (Green)
