# Supplementary material for: Upregulation of glycosaminoglycan synthesis by Neurotropin in nucleus pulposus cells via stimulation of chondroitin sulfate N-acetylgalactosaminyltransferase 1: A new approach to attenuation of intervertebral disc degeneration
Source: PLoS One. 2018 Aug 27;13(8):e0202640. doi: 10.1371/journal.pone.0202640 (PMC6110471; doi:10.1371/journal.pone.0202640)
Supplement: S1 Table — (PDF) [file pone.0202640.s002.pdf]

S3 Table

Functional analysis of the 697 genes upregulated higher than two-fold by NTP

Term	Count	P-Value
**GO:0045071 negative regulation of viral genome replication**	9	2.0E-04
**GO:0006955 immune response**	31	2.8E-03
**GO:0051607 defense response to virus**	16	3.6E-03
**GO:0060337 type I interferon signaling pathway**	9	4.8E-03
**GO:0009636 response to toxic substance**	9	2.4E-02
**GO:0042493 response to drug**	21	2.9E-02
**GO:0009615 response to virus**	10	3.9E-02
**GO:0034138 toll-like receptor 3 signaling pathway**	3	4.1E-02
**GO:0060054 positive regulation of epithelial cell proliferation involved in wound healing**	3	4.1E-02
**GO:0006805 xenobiotic metabolic process**	8	4.3E-02

Biological processes implicated in response to extraneous material are ordered in accordance to the attributed descriptive *p*-value
